# Bis(μ-biphenyl-2,2′-dicarboxyl­ato)bis­[(2,2′-bipyridine)cobalt(II)]

**DOI:** 10.1107/S1600536808037288

**Published:** 2008-11-20

**Authors:** Zhe An, Xian-Chun Niu

**Affiliations:** a School of Chemistry and Life Science, Maoming University, Maoming 525000, People’s Republic of China; b School of Chemical and Environmental Engineering, Maoming University, Maoming 525000, People’s Republic of China

## Abstract

In the title compound, [Co_2_(C_14_H_8_O_4_)_2_(C_10_H_8_N_2_)_2_], the Co^II^ atom is coordinated by two N atoms from one 2,2′-bipyridine ligand and two O atoms from two biphenyl-2,2′-dicarboxyl­ate (2,2′-dpa) ligands in a distorted planar geometry. Longer Co—O contacts [2.437 (3) and 2.552 (3) Å] are formed to the second O atom of each coordinated carboxyl­ate group so that these groups approximate a bidentate coordination mode and the coordination geometry around Co^II^ approaches distorted octa­hedral. The 2,2′-dpa ligands bridge two Co^II^ atoms, forming a cyclic dinuclear complex around a centre of inversion.

## Related literature

For metal-organic frameworks containing 2,2′-dpa, see: Rueff *et al.* (2003 ▶); Wang *et al.* (2006 ▶); Xu *et al.* (2006 ▶).
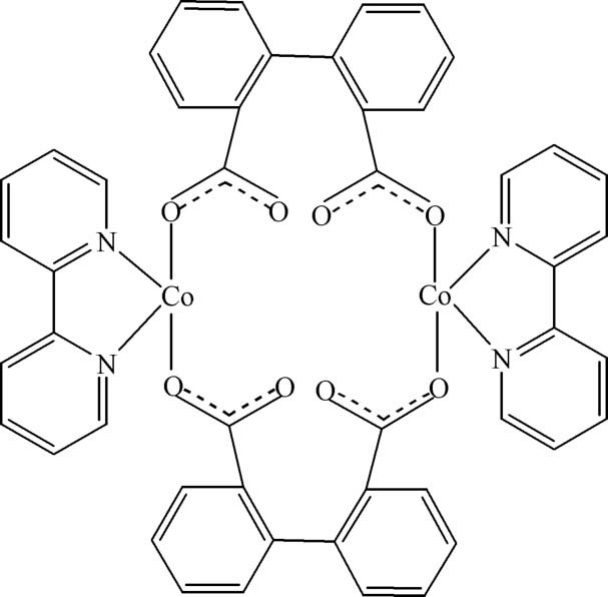


## Experimental

### 

#### Crystal data

[Co_2_(C_14_H_8_O_4_)_2_(C_10_H_8_N_2_)_2_]
*M* *_r_* = 910.64
Monoclinic, 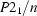

*a* = 11.236 (2) Å
*b* = 13.198 (2) Å
*c* = 13.393 (2) Å
β = 102.90 (2)°
*V* = 1936.1 (5) Å^3^
*Z* = 2
Mo *K*α radiation
μ = 0.92 mm^−1^
*T* = 296 (2) K
0.39 × 0.27 × 0.21 mm

#### Data collection

Bruker APEXII CCD diffractometer
Absorption correction: multi-scan (*SADABS*; Bruker, 2001 ▶) *T*_min_ = 0.715, *T*_max_ = 0.830
10081 measured reflections
3408 independent reflections
2591 reflections with *I* > 2σ(*I*)
*R*_int_ = 0.040

#### Refinement

*R*[*F* ^2^ > 2σ(*F* ^2^)] = 0.035
*wR*(*F* ^2^) = 0.093
*S* = 1.00
3408 reflections
280 parameters
H-atom parameters not refined
Δρ_max_ = 0.23 e Å^−3^
Δρ_min_ = −0.28 e Å^−3^

### 

Data collection: *APEX2* (Bruker, 2004 ▶); cell refinement: *SAINT-Plus* (Bruker, 2001 ▶); data reduction: *SAINT-Plus*; program(s) used to solve structure: *SHELXS97* (Sheldrick, 2008 ▶); program(s) used to refine structure: *SHELXL97* (Sheldrick, 2008 ▶); molecular graphics: *SHELXTL* (Sheldrick, 2008 ▶); software used to prepare material for publication: *SHELXTL*.

## Supplementary Material

Crystal structure: contains datablocks I, global. DOI: 10.1107/S1600536808037288/bi2316sup1.cif

Structure factors: contains datablocks I. DOI: 10.1107/S1600536808037288/bi2316Isup2.hkl

Additional supplementary materials:  crystallographic information; 3D view; checkCIF report

## Figures and Tables

**Fig. 1.**
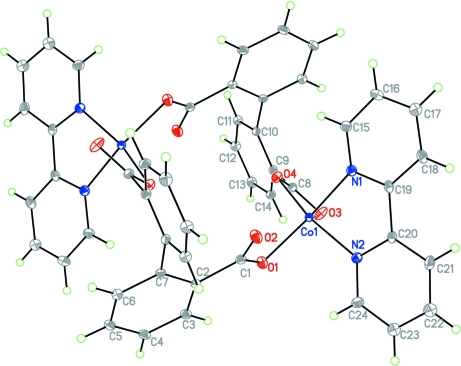
Molecular unit in the title compound showing displacement ellipsoids at 30% probability for non-H atoms. Non-labelled atoms are related to labelled atoms by the symmetry code: 1-x, 1-y, 1-z.

## supplementary crystallographic information

### Comment

biphenyl-2,2'-dicarboxylic acid (H_2_dpa) has been demonstrated to be a useful ligand for constructing metal-organic frameworks (Rueff *et al., *2003; Wang *et al., *2006; Xu *et al., *2006). The title compound is a cyclic dinuclear Co^II^ compound in which biphenyl-2,2'-dicarboxylate (2,2'-dpa) acts as a bridging ligand.

As shown in Figure 1, each Co^II^ atom is coordinated by two N atoms from one 2,2'-bipyridine ligand and two O atoms from two biphenyl-2,2'-dicarboxylate ligands, forming a distorted planar geometry. The Co—O and Co—N bond lengths are in the range 1.948 (2)–1.964 (2) and 1.968 (3)–1.989 (3) Å, respectively. Longer Co—O contacts (2.437 (3) and 2.552 (3) Å) are formed to the second O atom of each coordinated carboxylate group, so that these groups approximate a bidentate coordination mode and the coordination geometry around Co^II^ approaches distorted octahedral. The biphenyl-2,2'-dicarboxylate ligand acts as a bridge between Co^II^ atoms to form a cyclic dinuclear complex around a centre of inversion.

### Experimental

A mixture of cobalt(II) chloride hexahydrate (0.1 mmol), 2,2'-bipyridine (0.1 mmol), biphenyl-2,2'-dicarboxylic acid (0.2 mmol) and H_2_O (16 ml) in a 25 ml Teflon-lined stainless steel autoclave was kept at 463 K for five days. Red crystals were obtained after cooling to room temperature with a yield of 12%. Elemental analysis calculated: C 63.25, H 3.51, N 6.15%; found: C 63.21, H 3.39, N 6.09%.

### Refinement

H atoms were placed in calculated positions with C—H = 0.93 Å and refined as riding with *U*_iso_(H) = 1.2*U*_eq_(C).

### Crystal data

**Table:**

[Co_2_(C_14_H_8_O_4_)_2_(C_10_H_8_N_2_)_2_]	*F*_000_ = 932
*M**_r_* = 910.64	*D*_x_ = 1.562 Mg m^−^^3^
Monoclinic, *P*2_1_/*n*	Mo *K*α radiation λ = 0.71073 Å
Hall symbol: -P 2yn	Cell parameters from 3408 reflections
*a* = 11.236 (2) Å	θ = 2.1–25.0º
*b* = 13.198 (2) Å	µ = 0.92 mm^−^^1^
*c* = 13.393 (2) Å	*T* = 296 (2) K
β = 102.90 (2)º	Block, red
*V* = 1936.1 (5) Å^3^	0.39 × 0.27 × 0.21 mm
*Z* = 2	

### Data collection

**Table:**

Bruker APEXII CCD diffractometer	3408 independent reflections
Radiation source: fine-focus sealed tube	2591 reflections with *I* > 2σ(*I*)
Monochromator: graphite	*R*_int_ = 0.040
*T* = 296(2) K	θ_max_ = 25.0º
φ and ω scans	θ_min_ = 2.1º
Absorption correction: multi-scan(SADABS; Bruker, 2001)	*h* = −13→13
*T*_min_ = 0.715, *T*_max_ = 0.830	*k* = −15→15
10081 measured reflections	*l* = −15→7

### Refinement

**Table:**

Refinement on *F*^2^	Secondary atom site location: difference Fourier map
Least-squares matrix: full	Hydrogen site location: inferred from neighbouring sites
*R*[*F*^2^ > 2σ(*F*^2^)] = 0.035	H-atom parameters not refined
*wR*(*F*^2^) = 0.093	*w* = 1/[σ^2^(*F*_o_^2^) + (0.049*P*)^2^ + 0.1211*P*] where *P* = (*F*_o_^2^ + 2*F*_c_^2^)/3
*S* = 1.00	(Δ/σ)_max_ = 0.005
3408 reflections	Δρ_max_ = 0.23 e Å^−^^3^
280 parameters	Δρ_min_ = −0.28 e Å^−^^3^
Primary atom site location: structure-invariant direct methods	Extinction correction: none

### Special details

**Table:**

Geometry. All esds (except the esd in the dihedral angle between two l.s. planes) are estimated using the full covariance matrix. The cell esds are taken into account individually in the estimation of esds in distances, angles and torsion angles; correlations between esds in cell parameters are only used when they are defined by crystal symmetry. An approximate (isotropic) treatment of cell esds is used for estimating esds involving l.s. planes.
Refinement. Refinement of F^2^ against ALL reflections. The weighted R-factor wR and goodness of fit S are based on F^2^, conventional R-factors R are based on F, with F set to zero for negative F^2^. The threshold expression of F^2^ > 2sigma(F^2^) is used only for calculating R-factors(gt) etc. and is not relevant to the choice of reflections for refinement. R-factors based on F^2^ are statistically about twice as large as those based on F, and R- factors based on ALL data will be even larger.

### Fractional atomic coordinates and isotropic or equivalent isotropic displacement parameters (Å^2^)

**Table:**

	*x*	*y*	*z*	*U*_iso_*/*U*_eq_	
Co1	0.69884 (3)	0.33942 (2)	0.55976 (3)	0.0362 (8)	
C1	0.4768 (3)	0.3112 (2)	0.5772 (2)	0.0376 (7)	
C2	0.3402 (2)	0.30053 (19)	0.5493 (2)	0.0340 (6)	
C3	0.2858 (3)	0.2453 (2)	0.4630 (2)	0.0432 (7)	
H3	0.3352	0.2114	0.4268	0.052*	
C4	0.1603 (3)	0.2389 (2)	0.4292 (2)	0.0479 (8)	
H4	0.1263	0.2007	0.3715	0.057*	
C5	0.0866 (3)	0.2894 (2)	0.4814 (2)	0.0466 (8)	
H5	0.0021	0.2864	0.4591	0.056*	
C6	0.1389 (3)	0.3447 (2)	0.5672 (2)	0.0420 (7)	
H6	0.0882	0.3789	0.6021	0.050*	
C7	0.2648 (2)	0.35125 (19)	0.6036 (2)	0.0341 (6)	
C8	0.7126 (2)	0.4230 (2)	0.3940 (2)	0.0368 (7)	
C9	0.6933 (2)	0.4851 (2)	0.2986 (2)	0.0344 (6)	
C10	0.6914 (2)	0.5897 (2)	0.2997 (2)	0.0331 (6)	
C11	0.6643 (3)	0.6406 (2)	0.2068 (2)	0.0427 (7)	
H11	0.6649	0.7111	0.2066	0.051*	
C12	0.6363 (3)	0.5892 (2)	0.1142 (2)	0.0483 (8)	
H12	0.6156	0.6251	0.0530	0.058*	
C13	0.6393 (3)	0.4859 (2)	0.1133 (2)	0.0477 (8)	
H13	0.6215	0.4506	0.0516	0.057*	
C14	0.6691 (3)	0.4344 (2)	0.2050 (2)	0.0417 (7)	
H14	0.6732	0.3641	0.2044	0.050*	
C15	0.8778 (3)	0.4946 (2)	0.6591 (2)	0.0466 (8)	
H15	0.8341	0.5399	0.6116	0.056*	
C16	0.9763 (3)	0.5291 (3)	0.7318 (3)	0.0563 (9)	
H16	0.9998	0.5967	0.7320	0.068*	
C17	1.0395 (3)	0.4634 (3)	0.8038 (3)	0.0573 (9)	
H17	1.1048	0.4862	0.8543	0.069*	
C18	1.0053 (3)	0.3639 (3)	0.8004 (2)	0.0515 (8)	
H18	1.0474	0.3180	0.8481	0.062*	
C19	0.9069 (2)	0.3323 (2)	0.7249 (2)	0.0395 (7)	
C20	0.8635 (2)	0.2279 (2)	0.7100 (2)	0.0384 (7)	
C21	0.9169 (3)	0.1475 (3)	0.7677 (3)	0.0558 (9)	
H21	0.9813	0.1576	0.8238	0.067*	
C22	0.8741 (3)	0.0524 (3)	0.7416 (3)	0.0638 (10)	
H22	0.9100	−0.0032	0.7792	0.077*	
C23	0.7776 (3)	0.0393 (2)	0.6593 (3)	0.0611 (10)	
H23	0.7492	−0.0255	0.6396	0.073*	
C24	0.7238 (3)	0.1219 (2)	0.6068 (3)	0.0512 (8)	
H24	0.6560	0.1132	0.5531	0.061*	
N1	0.8438 (2)	0.39788 (17)	0.65536 (17)	0.0389 (6)	
N2	0.7664 (2)	0.21450 (17)	0.63096 (18)	0.0400 (6)	
O1	0.53335 (17)	0.28601 (15)	0.50773 (15)	0.0449 (5)	
O2	0.53139 (18)	0.34378 (16)	0.66074 (17)	0.0561 (6)	
O3	0.7602 (2)	0.33887 (15)	0.39642 (18)	0.0615 (7)	
O4	0.67220 (18)	0.45631 (14)	0.46882 (15)	0.0419 (5)	

### Atomic displacement parameters (Å^2^)

**Table:**

	*U*^11^	*U*^22^	*U*^33^	*U*^12^	*U*^13^	*U*^23^
Co1	0.0383 (19)	0.0317 (18)	0.0364 (19)	0.0024 (15)	0.0038 (15)	−0.0010 (15)
C1	0.0442 (17)	0.0298 (15)	0.0383 (17)	−0.0005 (13)	0.0083 (15)	−0.0045 (13)
C2	0.0371 (15)	0.0266 (14)	0.0379 (17)	−0.0029 (12)	0.0076 (13)	−0.0027 (12)
C3	0.0447 (18)	0.0380 (17)	0.0477 (19)	−0.0056 (13)	0.0124 (15)	−0.0125 (14)
C4	0.0467 (18)	0.0432 (17)	0.050 (2)	−0.0131 (15)	0.0029 (16)	−0.0116 (15)
C5	0.0365 (16)	0.0453 (18)	0.054 (2)	−0.0079 (14)	0.0016 (16)	−0.0012 (16)
C6	0.0369 (16)	0.0431 (17)	0.0467 (19)	−0.0014 (14)	0.0106 (14)	−0.0031 (15)
C7	0.0364 (15)	0.0271 (14)	0.0370 (16)	−0.0039 (12)	0.0045 (13)	0.0022 (12)
C8	0.0388 (16)	0.0325 (16)	0.0370 (17)	0.0033 (13)	0.0040 (14)	−0.0015 (13)
C9	0.0343 (15)	0.0353 (15)	0.0324 (16)	0.0019 (12)	0.0050 (13)	0.0012 (13)
C10	0.0306 (14)	0.0333 (15)	0.0347 (16)	0.0017 (12)	0.0060 (13)	−0.0009 (13)
C11	0.0490 (18)	0.0375 (16)	0.0417 (18)	0.0070 (13)	0.0103 (15)	0.0072 (14)
C12	0.0558 (19)	0.055 (2)	0.0330 (17)	0.0089 (16)	0.0067 (15)	0.0071 (15)
C13	0.0503 (18)	0.058 (2)	0.0329 (17)	0.0039 (16)	0.0044 (15)	−0.0060 (15)
C14	0.0448 (17)	0.0371 (16)	0.0405 (18)	0.0011 (13)	0.0041 (15)	−0.0048 (14)
C15	0.0478 (18)	0.0430 (18)	0.0472 (19)	−0.0048 (15)	0.0067 (16)	0.0030 (15)
C16	0.054 (2)	0.056 (2)	0.058 (2)	−0.0192 (17)	0.0091 (18)	−0.0071 (18)
C17	0.0416 (18)	0.078 (3)	0.048 (2)	−0.0164 (18)	0.0013 (16)	−0.0016 (19)
C18	0.0378 (17)	0.070 (2)	0.0420 (19)	−0.0044 (16)	−0.0002 (15)	0.0102 (16)
C19	0.0322 (15)	0.0532 (18)	0.0326 (16)	0.0027 (14)	0.0059 (13)	0.0054 (14)
C20	0.0358 (15)	0.0466 (17)	0.0337 (16)	0.0079 (13)	0.0099 (14)	0.0081 (14)
C21	0.0496 (19)	0.059 (2)	0.057 (2)	0.0149 (17)	0.0072 (17)	0.0198 (17)
C22	0.061 (2)	0.051 (2)	0.082 (3)	0.0163 (18)	0.023 (2)	0.028 (2)
C23	0.068 (2)	0.0381 (19)	0.079 (3)	0.0010 (17)	0.021 (2)	0.0111 (18)
C24	0.057 (2)	0.0408 (18)	0.056 (2)	−0.0040 (16)	0.0150 (17)	0.0012 (16)
N1	0.0384 (13)	0.0417 (15)	0.0352 (14)	0.0009 (11)	0.0056 (11)	0.0031 (11)
N2	0.0421 (14)	0.0380 (14)	0.0397 (15)	0.0009 (11)	0.0087 (12)	0.0045 (11)
O1	0.0396 (11)	0.0581 (14)	0.0366 (12)	0.0006 (10)	0.0072 (10)	−0.0038 (10)
O2	0.0415 (12)	0.0719 (15)	0.0515 (14)	−0.0037 (11)	0.0030 (11)	−0.0269 (12)
O3	0.0940 (18)	0.0411 (13)	0.0539 (14)	0.0263 (12)	0.0263 (14)	0.0070 (11)
O4	0.0513 (12)	0.0396 (11)	0.0350 (11)	0.0073 (9)	0.0101 (10)	0.0016 (9)

### Geometric parameters (Å, °)

**Table:**

Co1—O4	1.9469 (18)	C11—H11	0.930
Co1—O1	1.9651 (19)	C12—C13	1.365 (4)
Co1—N2	1.970 (2)	C12—H12	0.930
Co1—N1	1.989 (2)	C13—C14	1.377 (4)
Co1—C8	2.514 (3)	C13—H13	0.930
C1—O2	1.228 (3)	C14—H14	0.930
C1—O1	1.281 (3)	C15—N1	1.330 (4)
C1—C2	1.503 (4)	C15—C16	1.378 (4)
C2—C3	1.388 (4)	C15—H15	0.930
C2—C7	1.404 (4)	C16—C17	1.372 (4)
C3—C4	1.384 (4)	C16—H16	0.930
C3—H3	0.930	C17—C18	1.366 (4)
C4—C5	1.370 (4)	C17—H17	0.930
C4—H4	0.930	C18—C19	1.385 (4)
C5—C6	1.378 (4)	C18—H18	0.930
C5—H5	0.930	C19—N1	1.351 (3)
C6—C7	1.392 (4)	C19—C20	1.461 (4)
C6—H6	0.930	C20—N2	1.351 (3)
C7—C10^i^	1.497 (4)	C20—C21	1.370 (4)
C8—O3	1.230 (3)	C21—C22	1.360 (5)
C8—O4	1.267 (3)	C21—H21	0.930
C8—C9	1.493 (4)	C22—C23	1.373 (5)
C9—C10	1.380 (4)	C22—H22	0.930
C9—C14	1.393 (4)	C23—C24	1.364 (4)
C10—C11	1.387 (4)	C23—H23	0.930
C10—C7^i^	1.497 (4)	C24—N2	1.326 (4)
C11—C12	1.386 (4)	C24—H24	0.930
			
O4—Co1—O1	93.51 (8)	C13—C12—H12	120.1
O4—Co1—N2	162.73 (9)	C11—C12—H12	120.1
O1—Co1—N2	95.93 (9)	C12—C13—C14	119.1 (3)
O4—Co1—N1	94.86 (9)	C12—C13—H13	120.5
O1—Co1—N1	160.30 (9)	C14—C13—H13	120.5
N2—Co1—N1	80.88 (9)	C13—C14—C9	121.7 (3)
O4—Co1—C8	29.68 (8)	C13—C14—H14	119.2
O1—Co1—C8	94.82 (9)	C9—C14—H14	119.2
N2—Co1—C8	134.56 (9)	N1—C15—C16	121.5 (3)
N1—Co1—C8	101.27 (9)	N1—C15—H15	119.2
O2—C1—O1	121.7 (3)	C16—C15—H15	119.2
O2—C1—C2	122.2 (3)	C17—C16—C15	119.7 (3)
O1—C1—C2	116.1 (2)	C17—C16—H16	120.1
C3—C2—C7	118.5 (2)	C15—C16—H16	120.1
C3—C2—C1	119.5 (3)	C18—C17—C16	119.1 (3)
C7—C2—C1	121.9 (2)	C18—C17—H17	120.5
C4—C3—C2	122.2 (3)	C16—C17—H17	120.5
C4—C3—H3	118.9	C17—C18—C19	119.1 (3)
C2—C3—H3	118.9	C17—C18—H18	120.4
C5—C4—C3	119.4 (3)	C19—C18—H18	120.4
C5—C4—H4	120.3	N1—C19—C18	121.4 (3)
C3—C4—H4	120.3	N1—C19—C20	113.7 (2)
C4—C5—C6	119.3 (3)	C18—C19—C20	124.8 (3)
C4—C5—H5	120.3	N2—C20—C21	121.1 (3)
C6—C5—H5	120.3	N2—C20—C19	114.6 (2)
C5—C6—C7	122.4 (3)	C21—C20—C19	124.3 (3)
C5—C6—H6	118.8	C22—C21—C20	119.0 (3)
C7—C6—H6	118.8	C22—C21—H21	120.5
C6—C7—C2	118.2 (3)	C20—C21—H21	120.5
C6—C7—C10^i^	116.6 (3)	C21—C22—C23	119.5 (3)
C2—C7—C10^i^	125.2 (2)	C21—C22—H22	120.2
O3—C8—O4	121.5 (3)	C23—C22—H22	120.2
O3—C8—C9	119.8 (3)	C24—C23—C22	119.5 (3)
O4—C8—C9	118.5 (2)	C24—C23—H23	120.2
O3—C8—Co1	72.13 (17)	C22—C23—H23	120.2
O4—C8—Co1	49.52 (13)	N2—C24—C23	121.1 (3)
C9—C8—Co1	166.2 (2)	N2—C24—H24	119.5
C10—C9—C14	119.3 (3)	C23—C24—H24	119.5
C10—C9—C8	122.7 (3)	C15—N1—C19	119.1 (3)
C14—C9—C8	117.9 (2)	C15—N1—Co1	125.7 (2)
C9—C10—C11	118.4 (3)	C19—N1—Co1	115.06 (19)
C9—C10—C7^i^	121.8 (2)	C24—N2—C20	119.7 (3)
C11—C10—C7^i^	119.2 (2)	C24—N2—Co1	125.0 (2)
C12—C11—C10	121.7 (3)	C20—N2—Co1	115.23 (18)
C12—C11—H11	119.1	C1—O1—Co1	103.46 (17)
C10—C11—H11	119.1	C8—O4—Co1	100.80 (16)
C13—C12—C11	119.7 (3)		

Symmetry codes: (i) −*x*+1, −*y*+1, −*z*+1.
